# Testicular Sarcoidosis: Diagnostic Approach and Management Strategies

**DOI:** 10.7759/cureus.12715

**Published:** 2021-01-15

**Authors:** Brice Ballet, Marc Roelandt, Fabrice Lockefeer, David Thüer

**Affiliations:** 1 Faculty of Medicine and Health Sciences, University of Antwerp, Antwerp, BEL; 2 Respiratory Medicine, AZ Monica, Antwerp, BEL; 3 Pathology, AZ Monica, Antwerp, BEL; 4 Urology, AZ Monica, Antwerp, BEL

**Keywords:** sarcoidosis, testicular mass

## Abstract

Sarcoidosis is a multisystem disorder which, in rare cases, can affect the urogenital tract. The clinical presentation of this benign inflammatory disorder can easily mimic that of testicular malignancy. Therefore, it is crucial to differentiate between these two entities, as misdiagnosis may lead to unnecessary surgical interventions, which have important implications for future fertility. While testicular cancer must always be ruled out, sarcoidosis should be considered in all patients presenting with a testicular mass. Here, we present a case of sarcoidosis with bilateral epididymal and testicular involvement. The diagnosis was made by frozen section and the patient was treated with corticosteroids.

## Introduction

Sarcoidosis is a systemic inflammatory disorder of unknown etiology, characterized histologically by the presence of noncaseating granulomas in affected tissues. It is predominantly a disorder of the lungs but can affect most of the body with ophthalmological, dermatological, endocrine and reproductive manifestations. Urogenital involvement with sarcoidosis is rare, with only 0.2% of patients diagnosed clinically and 5% post-mortem [1]. The kidney is the most common urogenital organ affected, followed by the epididymis, testis and vas deferens [2]. The peak incidence of sarcoidosis and testicular cancer coincide at 20 to 40 years, which is why many patients with testicular sarcoidosis end up having an unnecessary orchiectomy or epididymectomy [3].

## Case presentation

**Figure 1:**
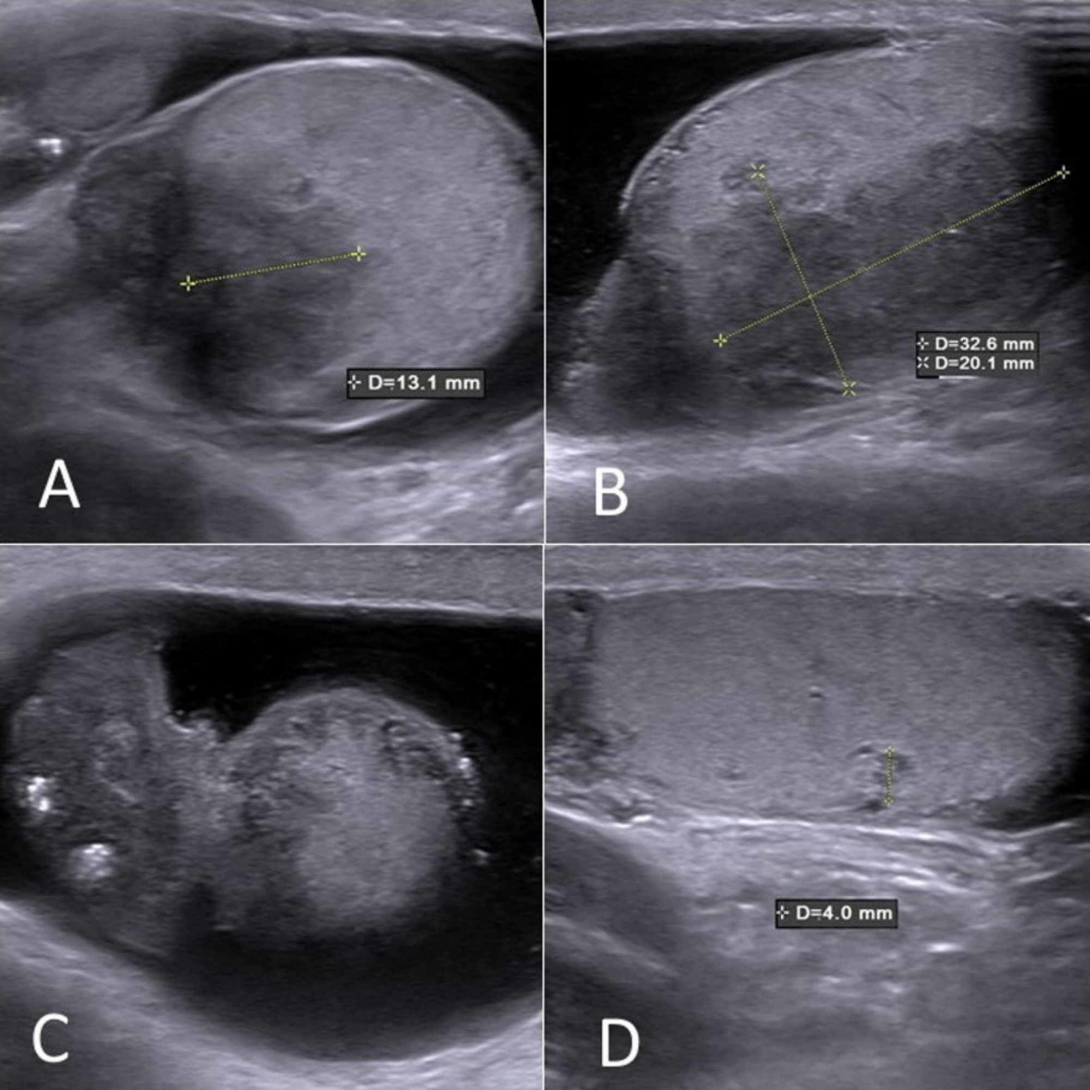
Scrotal ultrasound Bilateral hypoechogenic nodules in both epididymal heads (A, B), and a right-sided testicular infiltration along the rete testis (C). A small parenchymal lesion was noted in the right testis (D).

**Figure 2:**
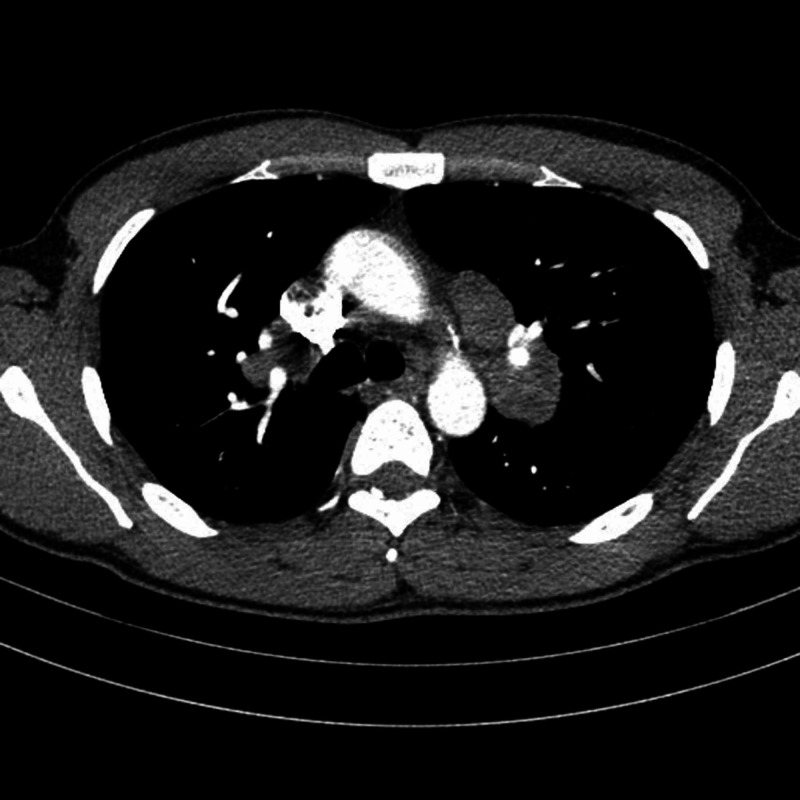
CT thorax revealing bilateral hilar lymphadenopathy

A 32-year-old black male was presented with a one-month history of painful swelling in the right hemiscrotum not responding to initial antibiotic therapy. Past medical history was unremarkable and there was no family history of testicular cancer or systemic disease. On examination, he had bilateral epididymal enlargement and tenderness of the right hemiscrotum. Scrotal ultrasound revealed bilateral hypoechogenic nodules of approximately 1 cm in both epididymal heads, with a right-sided testicular infiltration along the rete testis. Additionally, a small parenchymal lesion of approximately 3 mm was noted in the right testis (Figure 1). Computed tomography (CT) scan of the thorax, abdomen and pelvis revealed bilateral hilar lymphadenopathy, but no retroperitoneal lymphadenopathy or lung parenchymal involvement (Figure 2). Subsequently, endobronchial ultrasound (EBUS) guided biopsy of mediastinal lymph nodes showed noncaseating granulomas with a negative Ziehl-Neelsen stain for acid-fast bacilli. Angiotensin-converting enzyme (ACE) was elevated at 92 U/L (normal range 15-70 U/L). Serum calcium, C-reactive protein (CRP) and white blood cell count were normal. Testicular tumor markers alpha-fetoprotein (AFP), beta-human chorionic gonadotrophin (β-HCG) and lactate dehydrogenase (LDH) were also within the normal range. Semen analysis showed azoospermia.

**Figure 3:**
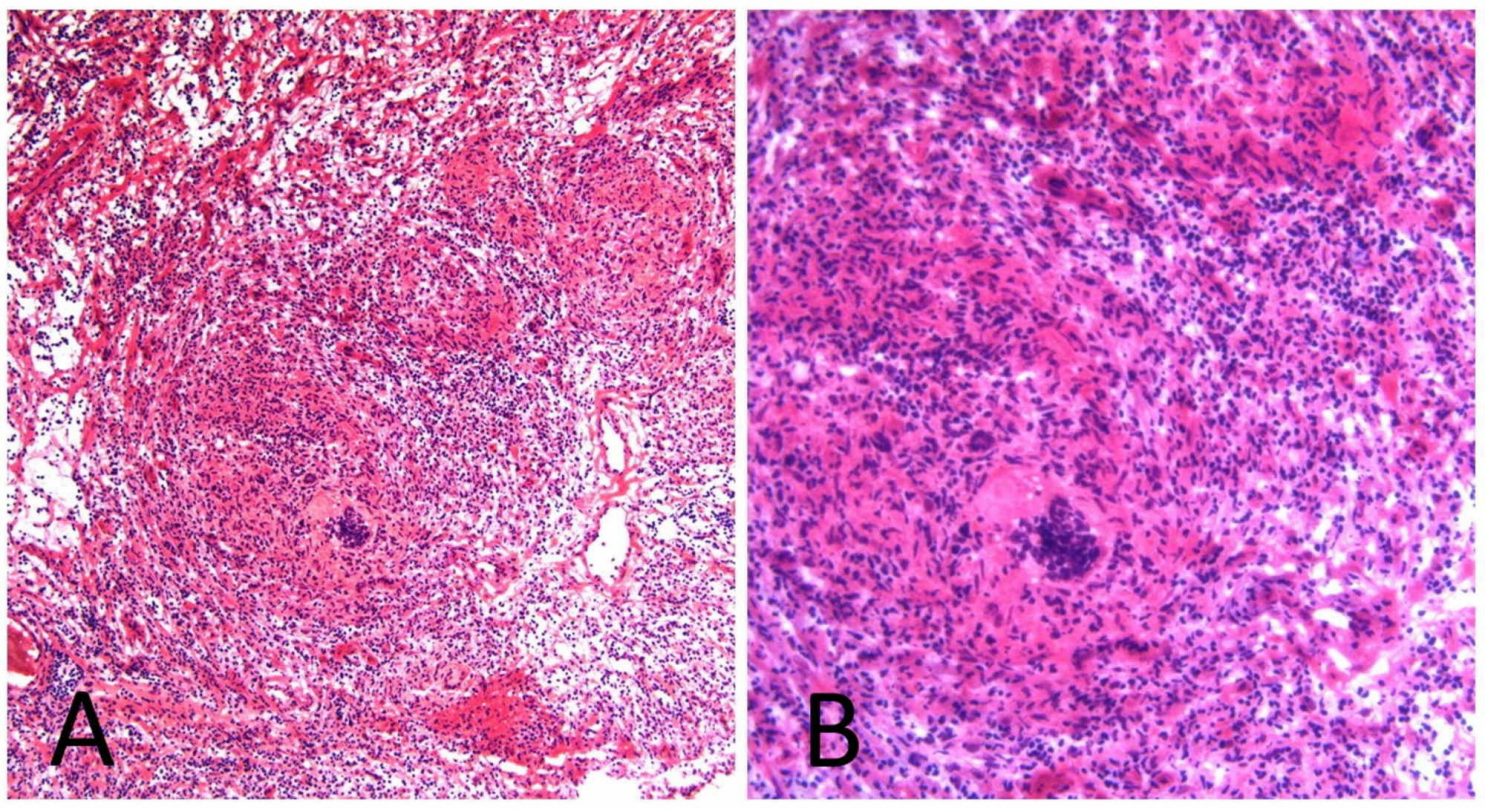
Histology slides showing characteristic granulomatous epididymitis Low (A) and high (B) magnification.

The patient disappeared from care and returned to our practice after six months complaining of increased scrotal soreness and swelling. Further enlargement of the epididymal and testicular lesions and consecutive loss of parenchymal tissue was detected on ultrasound, and the patient was referred for a scrotal exploration by inguinal approach. A frozen section of testicular and epididymal tissue confirmed the presence of non-necrotizing granulomatous disease, consistent with sarcoidosis (Figure 3). There were no histological signs of testicular neoplasia. Ziehl-Neelsen stain, tuberculosis and fungal culture came back negative.

Based on the progressive nature of the granulomatous inflammation, the patient’s increasing discomfort and desire to have children, corticosteroid therapy was started. Hence, the patient was treated on a tapered schedule of methylprednisolone, starting with 32 mg per day for two weeks, followed by 24 mg per day for two weeks, 16 mg per day for two weeks, 8 mg per day for two weeks, reaching a maintenance dose of 8 mg every other day. The patient discontinued the medication on his own initiative one month after the maintenance dose was achieved. Response to therapy was excellent, with complete resolution of symptoms and regression of the epididymal and testicular lesions on ultrasound. Genital tract patency, however, was not restored, as semen analysis showed persistent azoospermia.

## Discussion

Sarcoidosis is a multisystem inflammatory disorder most commonly found in the lungs and lymphatic system. In autopsy series, sarcoidosis of the urogenital tract is seen in 5% of patients. In the majority of these cases, involvement is limited to the kidneys. To a lesser extent, the involvement of the epididymis and testis can be seen [1]. Over 50% of urogenital sarcoidosis is found in black men, with less than 10% of cases in Caucasian men [3]. It can present as a painless scrotal mass or can mimic acute epididymo-orchitis, and can be accompanied by nephrocalcinosis (secondary to hypercalcemia) and systemic manifestations such as hilar adenopathy and erythema nodosum.

Following the identification of a scrotal mass, the differential diagnosis should include other causes of granulomatous disease such as tuberculosis and fungal diseases. Testicular cancer is the leading differential that must be excluded since its peak incidence coincides with sarcoidosis at 20 to 40 years of age. Generally, testicular cancer is more common among Caucasian males, while sarcoidosis mainly affects black males [4]. However, despite a lower prevalence in the black population, a high index of suspicion for malignancy must be maintained regardless of the patient’s race.

Ultrasound can be helpful in differentiating sarcoidosis from testicular cancer: involvement of the epididymis, especially if bilateral, makes a neoplastic etiology less likely. Sarcoidosis should therefore be considered in cases of multiple masses that simultaneously affect the epididymis and testes. Serum markers should always be determined in the work-up of a testicular mass: in our case, elevated ACE and normal levels of tumor markers (AFP, B-HCG) pointed towards a diagnosis of sarcoidosis. Lastly, a biopsy of skin lesions or endoscopic ultrasound biopsy of mediastinal lymph nodes can lead to a more definitive diagnosis and can be invaluable in differentiating pulmonary sarcoidosis from metastasized testicular carcinoma.

The unclear relationship between malignancy and sarcoidosis has been a cause for much debate. A study by Rayson et al. among patients seen at the Mayo Clinic showed a 100-fold increased incidence of sarcoidosis after treatment for testicular cancer [5]. However, the regular radiological examinations give rise to access and surveillance bias, which should be considered in the interpretation of this increased risk. A literature review by Paparel et al. demonstrated that sarcoidosis was diagnosed concomitantly in 31% of cases with all types of testicular tumors. Sarcoidosis regressed spontaneously in 80% of cases and did not alter the management or the cancer prognosis [6]. Given this strong association between sarcoidosis and testicular carcinoma, long-term follow-up and screening of our patient may be necessary.

Considering the possible etiologic relationship between testicular cancer and sarcoidosis, all patients with a testicular lesion and sarcoidosis should be strongly encouraged to undergo an inguinal exploration with biopsy. Some authors advocate orchiectomy for all patients with unilateral masses, even if sarcoidosis is present in other organs [7]. By contrast, others consider prompt orchiectomy to be a quite drastic measure and believe that radical orchiectomy should be reserved for cases of diffuse involvement of a testis or indeterminate histology [8]. We believe that both are acceptable approaches, and that management should be tailored to the patient. At the minimum, the testis should be approached through an inguinal incision with ultrasound-guided biopsies to rule out malignancy. If the analysis of the preoperative frozen section reveals typical granulomas, the testis may be spared. In our case, in the absence of risk factors for malignancy, we decided to manage the patient conservatively to maintain his fertility.

Treatment of sarcoidosis is generally expectant, as most patients will see spontaneous resolution within two years [9]. However, if symptoms are severe or organ function is threatened, corticosteroid treatment should be initiated. For instance, azoospermia or oligospermia may arise from epididymal involvement of sarcoidosis. In these cases, preservation of fertility must be weighed against the risks of long-term systemic steroid use. Svetec et al. suggest all patients concerned with future fertility obtain a screening semen analysis at the time of diagnosis. In the case of oligospermia or evidence of epididymal involvement, sperm banking should be offered [10].

## Conclusions

When testicular masses are encountered in patients, the differential diagnosis must consider testicular sarcoidosis, particularly in patients with confirmed sarcoidosis or epididymal involvement. This case demonstrates the need for histological confirmation prior to orchiectomy as - in contrast to testicular cancer - testicular sarcoidosis may be managed conservatively in patients who wish to preserve their fertility. Lastly, health care providers should be aware of the possible association between testicular cancer and sarcoidosis; long-term follow-up of these patients may be necessary.
